# Monitoring travellers from Ebola-affected countries in New South Wales, Australia: what is the impact on travellers?

**DOI:** 10.1186/s12889-017-4016-2

**Published:** 2017-01-24

**Authors:** Jocelyn Chan, Mahomed Patel, Sean Tobin, Vicky Sheppeard

**Affiliations:** 1 Health Protection New South Wales (NSW), NSW Health, Locked Mail Bag 961 North, Sydney, NSW 2059 Australia; 2 National Centre for Epidemiology and Population Health (NCEPH), Australian National University, Canberra, ACT Australia

## Abstract

**Background:**

Amidst an Ebola virus disease (EVD) epidemic of unprecedented magnitude in west Africa, concerns about the risk of importing EVD led to the introduction of programs for the screening and monitoring of travellers in a number of countries, including Australia. Emerging reports indicate that these programs are feasible to implement, however rigorous evaluations are not yet available. We aimed to evaluate the program of screening and monitoring travellers in New South Wales.

**Methods:**

We conducted a mixed methods study to evaluate the program of screening and monitoring travellers in New South Wales. We extracted quantitative data from the Notifiable Conditions Information Management System database and obtained qualitative data from two separate surveys of public health staff and arrivals, conducted by phone.

**Results:**

Between 1 October 2014 and 13 April 2015, public health staff assessed a total of 122 out of 123 travellers. Six people (5%) developed symptoms compatible with EVD and required further assessment. None developed EVD. Aid workers required lower levels of support compared to other travellers. Many travellers experienced stigmatisation. Public health staff were successful in supporting travellers to recognise and manage symptoms.

**Conclusion:**

We recommend that programs for monitoring travellers should be tailored to the needs of different populations and include specific strategies to remediate stigmatisation.

## Background

During the Ebola virus disease (EVD) epidemic of unprecedented magnitude in West Africa, concerns of the risk of importation of EVD led a number of countries to introduce entry screening and monitoring of travellers from EVD affected areas; these countries included United Kingdom (UK) [1], United States of America (US) [2, 3], Japan [4], Israel [5], and Australia. Monitoring symptoms, with or without restricting movement of individuals who have been in contact with the disease is a well-established public health measure to contain outbreaks of EVD. However the application of these measures to travellers from EVD affected areas is relatively new and evidence of the effectiveness of these measures is limited.

While the World Health Organization (WHO) has not issued guidance on the need for monitoring travellers from EVD affected countries, it has provided guidelines for countries wanting to introduce entry screening [6]. The European Centers for Disease Prevention and Control (ECDC) recommends monitoring of healthcare workers returning from EVD affected areas but not for other travellers [7]. The US Centers for Disease Control and Prevention (CDC) recommends entry screening and follow-up monitoring for travellers from EVD affected countries, regardless of whether they had known contact with an EVD case because, “*travellers from countries with widespread transmission or uncertain control measures may be unaware of their exposure to individuals with symptomatic Ebola infection*” [3].

Emerging reports from countries that screened and monitored travellers indicate that these programs are feasible to implement. In the US, 10,344 persons in 60 jurisdictions were monitored between 3 November 2014 and 8 March 2015 [8]. In the UK, 3388 passengers were screened at airports between 14 November 2014 and 4 January 2015, and 130 people were referred for monitoring [9]. While the results of rigorous evaluations of such programs are not yet available, commentaries and descriptive studies have highlighted potential problems [4, 5, 10–12]. In Japan, a symptomatic traveller under monitoring sought healthcare at a local clinic without disclosing a travel history, highlighting a weakness of the system to direct symptomatic travellers to seek appropriate healthcare [4]. In Israel, two travellers evaded screening at the border and were therefore not monitored before they presented to a hospital with fever, highlighting the low sensitivity of the screening process [5]. In the US, a review of jurisdictional procedures for screening and monitoring indicated that 17 of 55 states and territories implemented more restrictive policies, such as quarantine, than recommended by the CDC [10]. Commentaries from other authors highlighted the potential for such restrictive policies to hinder responses to the epidemic [11, 12].

Concerns have also been raised about the potential that screening and monitoring could exacerbate stigmatisation of travellers from Ebola affected countries [13]. However no study has described what impact the monitoring process may have on travellers.

Our aim was to describe the program of screening and monitoring travellers from EVD affected countries in New South Wales (NSW), with a particular focus on the impact of these measures on the travellers.

## Methods

### Program description

The rationale for the program, which commenced on 1 October 2014, was to enable early identification of symptoms in travellers who may have had contact with EVD, to direct them to appropriate health care facilities, and to facilitate early management for better outcomes and to minimise further spread.

We used three mechanisms to identify travellers who had visited an EVD affected area within the 21 days preceding their arrival in NSW: 1) entry screening at airports and ports, 2) lists of returning aid workers from aid organisations that had recruited them and, 3) lists of visas granted by the Department of Immigration and Border Protection to nationals from the three EVD affected countries. Lists of travellers identified through entry screening at Sydney International Airport were available from 14 November 2014; these travellers declared travel to EVD affected countries on a separate EVD-specific arrivals card. The latter two mechanisms were in place at the commencement of the program on 1 October 2014.

Based on intended place of residence, staff from the Communicable Disease Branch (CDB) at NSW Health distributed the contact information to one of 15 local public health units (PHUs) in NSW for follow-up who then contacted individuals within 24 h of arrival to conduct a more detailed risk assessment (criteria detailed in Table 1).

**Table 1:** Summary of exposure risk categories used by NSW Health (source: CDNA Series of National Guidelines on Ebola virus disease [22])

Category	Criteria
Very low risk	• Near vicinity of an EVD patient • Visiting a country with widespread EVD transmission in the past 21 days with no known exposures • Adequate PPE when in direct contact with EVD case in Australia
Low risk	• Household member of EVD case • Inadequate PPE plus close contact (being within 1 m of a EVD patient or within room for a prolonged period of time) • Inadequate PPE and brief direct contact (e.g. shaking hands) • Adequate PPE and direct contact if in an area of widespread EVD transmission
High risk	• Percutaneous (e.g. needle stick) or mucous membrane exposure to blood or body fluids of EVD patient • Inadequate PPE and direct skin contact exposure to blood or body fluids of EVD patient • Inadequate PPE and lab processing of body fluids of an EVD patient • Inadequate PPE and direct contact with deceased EVD patient or patient with unknown cause of death in an EVD affected area

All individuals were instructed to monitor their temperature and report to public health officials daily or weekly, depending on the risk level. Arriving travellers were also asked to report immediately any symptoms of interest (fever and other symptoms compatible with EVD including headache, vomiting or diarrhoea, myalgia, abdominal pain or unexplained bruising or bleeding) and to contact the PHU should they require any medical attention. Individuals reporting any of these symptoms were notified to the CDB who would decide on their management in consultation with an infectious diseases (ID) physician and the PHU. Individuals with high and low risk exposures were advised to minimise social mixing and travel, and to stay within ready access of appropriate health facilities. While quarantine was never recommended, isolation for limited periods was recommended while patients were symptomatic.

Public health unit staff conducted the risk assessment using standardised forms with data on demographics, travel history, potential exposures and any symptoms at initial assessment; the initial and follow-up data were recorded into the NSW Notifiable Conditions Information Management System (NCIMS).

### Study methods

This study comprised both qualitative and quantitative methods. We extracted data on the number of travellers, risk classifications, and identification and management of symptoms from the NCIMS database. We surveyed public health staff and travellers who had been screened and monitored to ascertain their experiences with the program, focusing on its impact on travellers. This survey was conducted by phone and collected data on experiences with three key elements of the program: (1) initial risk assessment and education, (2) monitoring of symptoms, and (3) recommendations on the restrictions of movement.

### Sampling

Quantitative data were extracted for all travellers from EVD affected countries screened between 1 October 2014 and 13 April 2015. For the qualitative component of the study, we identified a random stratified sample of travellers using a random number generator to sequence the travellers and interview sequentially until minimum quotas of 5 were reached in the two key demographic groups: aid workers and other travellers. Sample size was determined by the concept of saturation – we completed interviews until saturation of themes were reached [14]. One member of staff was interviewed from each public health unit that conducted monitoring.

### Data analysis

We obtained informed consent from all respondents and conducted thematic analysis of the transcripts of interviews to identify, group and report themes within the data [15].

## Results

### Travellers

Between 1 October 2014 and 13 April 2015, 123 travellers from EVD-affected countries were recorded on the NCIMS database, with a mean of 9 travellers per fortnight.

Of these, 57 (46%) were female and the median age was 39 years (range of 5 to 68 years). The most common reason for travel was aid work (*n* = 67, 55%) (28 were healthcare workers), followed by migrants from the affected areas (including humanitarian entry) (*n* = 23, 19%), other workers (*n* = 22, 18%) and leisure or visiting family members (*n* = 9, 7%). The category of aid work covered the fields of healthcare, public health, epidemiology, water and sanitation, logistics, and health promotion. Of those travelling for other types of work the most common reasons given were mining (*n* = 9, 41%) and media work (*n* = 6, 27%).

Of the 122 travellers who were assessed, 94 (77%) were in the very low risk category i.e. they had no known contact with any EVD cases. Twenty-eight (23%) were in the low risk category i.e. they had direct contact with EVD cases but had used appropriate personal protective equipment (PPE). None were in the high risk category i.e. direct contact with EVD cases without appropriate PPE.

No cases of EVD, either within or outside of our program, were identified in NSW during the study period. Six people developed symptoms compatible with EVD requiring further assessment: two people required brief admission to hospital for further investigation; two people were reviewed by ID physicians either as an outpatient or via telephone; and two people were reviewed by public health staff only (Table 2). Only one person was tested for EVD – the results were negative. Two other people developed unrelated symptoms requiring medical review (mental health assessment and urinary tract infection).

**Table 2:** Assessment and diagnosis for symptomatic travellers, New South Wales, October 2014 – April 2015

	Risk classification	Review	EVD test	Diagnosis
1	Very low	Local hospital	No	Influenza
2	Low	Designated hospital	Yes	Upper respiratory tract infection
3	Low	ID physician review	No	Non-specific symptoms
4	Very low	ID physician review	No	Adverse reaction to de-worming tablets
5	Low	Public health	No	Migraine
6	Very low	Public health	No	Isolated temperature

*ID physician* Infectious diseases physician

An additional arrival was transferred directly from the airport to the designated Viral Haemorrhagic Fever (VHF) hospital before entering the risk assessment and monitoring program; this person is not included in this report and did not develop EVD.

There were two travellers requiring hospitalisation. One was classified as low risk, was transferred to the designated VHF hospital the day after arrival with symptoms and was discharged for ongoing monitoring after testing negative for Ebola on polymerase chain reaction (PCR). The second traveller was classified as very low risk, was transferred to a local tertiary hospital and was not tested for EVD because this diagnosis was considered highly unlikely. The respiratory viral multiplex PCR was positive for influenza.

### Patient perspectives

Of the 24 travellers randomly sampled to participate in the study, 12 consented to be interviewed giving a response rate of 50%. The remaining 12 could not be contacted by phone, despite three attempts on consecutive days.

#### Impact

All travellers responded positively to the question on whether temperature monitoring measures were reasonable. Travellers described the process as simple, easy, and efficient.

Four interviewees (33%) had been advised to restrict movement. They reported these restrictions as being reasonable without any adverse social impacts, such as not being able to visit friends or attend church, and also financial impacts, with one person unable to go job-hunting during the monitoring period. Additionally, two travellers imposed their own restrictions to avoid being stigmatised by the community.

#### Personal relationships

Four travellers volunteered that they valued the personal contact and support from public health staff, describing them as ‘nice’ and ‘friendly’.

*“I felt supported rather than watched. It was like we were on the same team.”*

One arrival compared the personalised contact more favourably then the automated text messages received by a colleague in another state.

#### Different needs for different populations

Four aid workers reported they already had information about monitoring procedures in NSW from other colleagues or from their workplace. Three aid workers reported that they would have completed similar measures regardless of public health intervention as temperature monitoring was also instigated by employers and/or “ingrained” from time in the field. The most important element of the program for them was having the relevant contact details in the event of illness.

In contrast one arrival, a migrant, reported that the regular follow-up was useful to address questions or concerns that arose over time, especially relating to fear and misinformation that was widespread in the community. Another migrant responded that consistent follow-up was necessary as migrants may not have an understanding of the public health rationale for monitoring.

*“Most of us, we are not educated. If you don’t contact them, some will neglect to collect their body temperature… if they realise no-one will turn up, no-one will follow the system”*

Of the two interviewees who developed symptoms compatible with EVD, one was an aid worker who did not report the headache as it was an isolated symptom and, based on knowledge gained from his prior training, did not fulfil the case definition for EVD. Another case was in a migrant who reported fever with no other symptoms. He was advised to isolate himself from the other people in the family, take some paracetamol and monitor temperatures “more frequently”. The arrival described being grateful for the advice and felt reassured.

### Staff perspectives

A total of 12 staff from the 12 PHUs agreed to participate in the survey.

#### Good compliance

Staff members did not identify any major barriers to collection of self-monitoring data, and compliance was high after the initial contact.

*“Once or twice I had to remind them but they were always profusely apologetic. After all these people have lives.”*

Only two staff members described difficulty in obtaining the self-monitoring data. One described a healthcare worker, who was repeatedly late in providing the data and needed repeated prompting. Another person was lost to follow-up despite contact with family members who reported the person missing to the police. The person was later identified and had been asymptomatic.

#### Fear and stigmatisation

One staff member described a migrant who had actively evaded contact by providing false addresses. PHU staff successfully contacted this person after locating a friend who was able to reassure the contact that the PHU staff simply wanted to monitor their health. Another staff member described being rung up repeatedly by an irate member of the public using racist language about the presence of migrants from West Africa in their community. In collaboration with the migrant family, the PHU negotiated a mitigation strategy for the family to keep a low profile during the incubation period. Several PHUs (*n* = 3, 25%) provided assurances to schools, workplaces and medical facilities to enable travellers or their family members to attend school and work, and receive appropriate medical care.

#### Different needs for different populations

When asked about any difficulties with the risk assessment and education process, three PHUs (25%) responded that the process was easier because the majority of the travellers in their area were healthcare workers familiar with the disease and the principles and processes of monitoring.

## Discussion

All travellers that reported or developed symptoms compatible with EVD were managed appropriately. Management of some patients at home reduced the potential risks of transmission to the public and reduced the burden on the hospital system. There were no suspected or confirmed cases of EVD anywhere in NSW.

Although key elements of the program were agreed to by the Communicable Diseases Network Australia (CDNA) and implemented nationwide, implementation of this program was coordinated at a state level. This led to some jurisdictional differences, for example the Department of Health in Western Australia employed the use of an automated text messaging system [16]. The use of these systems may be suited to monitoring larger volumes of travellers [16]. As described in New York, the program in New South Wales relied on existing public health personnel, technology and systems [17].

While our study establishes the feasibility of monitoring in our setting, its feasibility in other settings will depend on the volume of travellers. The number of travellers in NSW were low compared with travellers in the US and UK, attributable to the smaller population and greater distance from West Africa. In the UK, over a similar 6-month period between 14 October 2014 and 7 April 2015, 6 031 travellers from EVD affected countries were screened at ports [9] and 470 of them were monitored by PHE [9]. In the US 10 344 persons were monitored in 60 states between November 3, 2014 and March 8, 2015 [8]. Similar to our experience in NSW, most travellers to US from EVD affected countries were in the low but not zero risk (91%) and a high proportion (99%) completed monitoring [8]. The need for, and potential value of these measures would depend on the context in which it is applied, with an assessment of the likely costs and benefits, i.e. the potential likelihood and impact of importation of the disease, the resources that can be mobilised for the purpose, and the need to manage community ‘outrage’ if such screening was not in place.

In NSW, five percent of travellers developed one or more symptoms compatible with EVD. This is higher than the one percent who developed symptoms from the 2 540 U.S. military service members monitored from 25 October 2014 to 27 February 2015 [18], and the one percent who developed symptoms from a population travellers in the US from 3 November 2014 to 8 March 2015 [8].

There was general support for the screening and monitoring program from travellers of all demographics and public health staff. Different levels of support were needed by the different categories of travellers. While migrants not familiar to our health systems appreciated ongoing communication over the monitoring period, travellers who were health workers were relatively more independent in monitoring temperatures and managing and reporting symptoms; the critical information they needed was how to access the health system appropriately.

Another theme identified was stigmatisation or fear of stigmatisation of travellers by the community. Similar attitudes and behaviours were reported in a number of countries. During the 2000 and 2001 EVD epidemics in Uganda for example, harassment, rejection, and abandonment of individuals with EVD were common occurrences. Similar reports were described in the West African epidemic [19, 20]. In the US, two household contacts of an EVD case stated they felt unsafe leaving their homes because of stigmatisation by others in their community after their photos, names, and addresses had been published in the media [21]. The evaluation demonstrated the positive role of public health authorities in mitigating some of the stigma surrounding travellers from EVD affected countries at the height of the epidemic. Public health authorities have an important role to play in remediating the stigma, by communicating to travellers and the wider public about real risks and appropriate preventative measures, as well as strategies to support individuals facing discrimination; this may include advocating on their behalf to other institutions, individual counselling and referral to social support services.

There are a number of limitations to this study. The primary evaluator participated in the development and implementation of the screening and monitoring program. This has the advantage of ensuring a thorough understanding of the program. To minimise potential biases, the design, methodology, results and recommendations of the evaluation were reviewed by supervisors external to NSW Health. Secondly this study focused on the monitoring process without examining the sensitivity of entry screening to identify travellers from EVD affected countries; this was because we did not have access to details of every arrival from West Africa from an alternative source. This may have implications for the representativeness of our results since travellers who avoided border screening may be less accepting of monitoring. However, all nationals from West African countries were identified and monitored based on the details provided to us of visas granted by the Department of Immigration and Border Protection. However the absence of travellers from EVD affected presenting to hospitals in NSW without having been previously monitored suggests that the entry screening process was successful in identifying at-risk travellers.

## Conclusion

In conclusion, screening and monitoring of travellers from EVD affected countries was feasible and widely accepted by travellers. Overall the program was successful in addressing the health and social needs of travellers, supporting travellers encountering stigmatisation and linking individuals with appropriate care in the event of illness. To increase its efficiency, resources should be directed towards supporting migrants in understanding the need for, and value of, the screening and monitoring activities, and for minimising their stigmatisation in the community. Relatively less supervision is required for returning health workers already familiar with the principles and importance of monitoring.

## Abbreviations

CDB: Communicable Diseases Branch, New South Wales Health
CDC: Centers for Disease Control and Prevention, United States of America
CDNA: Communicabel Diseases Network Australia
ECDC: European Centers for Disease Prevention and Control
EVD: Ebola virus disease
ID: Infectious diseases
NCIMS: Notifiable Conditions Information Management System
NSW: New South Wales, Australia
PCR: Polymerase chain reaction
PHU: Public Health Unit
PPE: Personal protective equipment
UK: United Kingdom
US: United States of America
VHF: Viral haemorrhagic fever
WHO: World Health Organization.
